# Adaptation, Marginal, and Occlusal Quality of Monolithic Zirconia Crowns: Effect of the CAD/CAM Milling Protocol

**DOI:** 10.1111/jerd.70181

**Published:** 2026-05-09

**Authors:** Indiarai Lavandoski Bringhenti, Andressa Restani Oliveira, Natalia Ulmi Ziglioli, Vitor de Trancoso de Britto, Alvaro Della Bona, Marcia Borba

**Affiliations:** ^1^ Graduate Program in Dentistry University of Passo Fundo Passo Fundo Brazil; ^2^ Division of Dentistry School of Medical Sciences, FBMH, University of Manchester Manchester UK

**Keywords:** adaptation, CAD/CAM, digital dentistry, fixed dental prosthesis, zirconia

## Abstract

**Objectives:**

Variables related to CAD/CAM processing can affect the final quality of all‐ceramic crowns. This study aims to evaluate the effect of the milling protocol on the adaptation, marginal and occlusal quality of monolithic zirconia crowns.

**Materials and Methods:**

Thirty‐nine monolithic zirconia crowns were produced using three CAD/CAM milling protocols (*n* = 13): slow (S), normal (N), and fast (F). Adaptation (gap thickness) was evaluated using the replica technique, while marginal quality was assessed according to a severity scale. Occlusal quality was investigated qualitatively, using a stereomicroscope, and quantitatively, through an occlusal dimensional discrepancy analysis. Gap thickness and occlusal discrepancy data were analyzed with ANOVA and Tukey's test, and marginal quality with Kruskal–Wallis and Student–Newman–Keuls (*α* = 0.05).

**Results:**

Gap thickness in the marginal, gingival‐axial angle and axial regions was similar among groups. In the axio‐occlusal angle and occlusal region, group N presented the smallest gap. For marginal quality, group F had higher scores in the severity scale than groups N and S for the mesial and buccal regions. Group F crowns showed less refined occlusal anatomy. Yet, when crowns produced with S and F protocols were compared with N, the total mean discrepancy was similar.

**Conclusion:**

The CAD/CAM milling protocol affected the adaptation, marginal, and occlusal quality of monolithic zirconia crowns.

## 1 Introduction

Polycrystalline zirconia ceramic has been widely used in prosthetic and implant dentistry due to its good aesthetics, biocompatibility, and outstanding mechanical properties [1, 2]. In addition, modifications were made to the composition and microstructure of zirconia polycrystals aiming for a more translucent material to produce monolithic dental restorations [1, 2, 3]. Second‐generation 3 mol% yttria partially stabilized zirconia (3Y‐PSZ) was developed by sintering at higher temperatures to increase the material's density and by reducing the concentration of the alumina additive. These modifications lead to an improvement in translucency when compared to first‐generation zirconia while preserving its high mechanical properties [1, 2, 3, 4, 5, 6, 7].

Zirconia dental restorations are frequently produced with the computer‐aided design/computer‐aided manufacturing (CAD/CAM) technology, which involves image acquisition (i.e., extra or intra‐oral scanning), restoration design, and manufacturing using subtractive (machining) or additive (3D‐printing) methods [8, 9, 10, 11, 12, 13]. In the past years, CAD software capabilities were restricted to defining the restoration anatomy and cement layer thickness, adjusting the occlusion, and choosing the type of restorative material [9]. More recently, the CAD software has been improved so that the operator can choose from different milling protocols [14, 15]. A fast‐milling protocol could produce a zirconia monolithic crown in only 12 min, while a slow protocol could create smoother and more refined anatomical surfaces at the expense of a longer processing time [15].

As the milling process can be tailored according to the treatment demand, new variables are included in the manufacturing process, which may affect the quality of the final restoration [14, 15, 16, 17, 18, 19, 20]. Several factors can affect the restoration accuracy and surface characteristics, including the number of axes of the CAM unit, the size and abrasiveness of the cutting tools (i.e., burs); the milling speed, pressure, and path; and the type of restorative material [12, 14, 15, 16, 17, 18, 19, 20, 21]. Moreover, polycrystalline zirconia can be milled in a partially or fully sintered state, in humid or dry conditions, which also influences the final outcome [14].

Adaptation and marginal quality are important aspects for the clinical success of prosthetic restorations [22, 23, 24, 25, 26]. The adaptation between the abutment and the ceramic crown in the occlusal and axial areas can affect the structural mechanical behavior [27, 28]. Greater gap thickness in the occlusal area was associated with a greater concentration of tensile stresses in the intaglio surface of ceramic crowns [27], while smaller gap thickness in the axial walls may induce hoop stresses [28]. In addition, large marginal gaps result in greater exposure of the cementation agents to oral fluids, enhancing the risk of material degradation. Lack of marginal integrity can also favor food impaction and biofilm formation [22, 23]. This combination of factors could lead to endodontic and periodontal biological complications.

Variables related to CAD/CAM processing can affect the adaptation and marginal quality of ceramic crowns [12, 17, 19, 20, 29, 30]. Margin defects can be introduced by the milling process and become zones of high stress concentration when the crown is subjected to loading in the oral environment [22, 31, 32]. A study reported that zirconia crowns with greater quantity and severity of margin defects had lower fracture load, with failures starting at the cervical margin of the crowns, precisely where the marginal defects were found [20].

Restorations with anatomically accurate, smooth, and well‐refined occlusal surfaces are desirable as they provide comfort to the patient, aesthetics, and reduced wear capacity [33, 34, 35]. Studies indicated that milling restorations with small and deep concave‐shaped details, as the ones observed in the occlusal surfaces, could be limited by the size and shape of the milling tools [13, 19]. Rough surfaces may cause premature occlusal contact and require extensive adjustment and polishing protocols. Especially for zirconia ceramics, adjustments with diamond burs are not recommended as they can generate surface and sub‐surface damage and micro‐cracks [4, 36]. Additionally, polishing can be very challenging due to the great hardness and fracture toughness of 3Y‐PSZ [1, 34, 35].

Therefore, considering the lack of information regarding the quality of dental prosthesis produced with these novel CAD/CAM milling protocols, the objective of this study was to evaluate the effect of the milling protocol (slow, normal, and fast) on the adaptation, marginal and occlusal quality of monolithic zirconia crowns. The study hypotheses are that the milling protocol affects the zirconia crowns' (1) gap thickness, (2) marginal quality, and (3) occlusal quality (anatomical characteristics and dimensional discrepancy).

## 2 Materials and Methods

Monolithic 3 mol% yttria partially stabilized zirconia (3Y‐PSZ; VITA YZ HT White, VITA Zahnfabrik, Bad Säckingen, Germany) crowns were produced using three different milling protocols (*n* = 13) and evaluated for adaptation (gap thickness), marginal quality (severity scale), and occlusal quality (anatomical characteristics and occlusal dimensional discrepancy). The sample size was calculated using the G*Power 3.1.9.4 software and the adaptation data from a pilot study, considering the following parameters: a = 0.05; power = 0.80; effect size = 0.55, number of groups = 3.

### 2.1 Specimen Preparation

A dentin‐analog (glass‐fiber reinforced epoxy resin, G10, Jiujiang Xinxing Insulation Material Co., Jiujiang City, China) rod was milled to simulate a simplified preparation for a maxillary premolar crown. The abutment preparation was 8 mm in diameter, 6 mm in height, had a total occlusal convergence angle of 12°, and all transitions between the axial and occlusal walls were smooth, rounded, and homogeneous. The preparation had a chamfer finish line and a radius of 1.2 mm between the cervical area and axial wall [10, 11, 28].

The abutment was inserted in a master model with adjacent teeth to simulate the clinical condition and was duplicated in a special type IV plaster. The plaster model was scanned using an extraoral scanner (InEos X5, Dentsplay Sirona, Bensheim, Germany). The digital model was used to design the maxillary second premolar monolithic crowns using the inLab CAM 19.0 software (Dentsply Sirona, Bensheim, Germany), with an 80 μm cement space [11, 15]. The same abutment was used to produce the crowns and to measure the gap thickness.

Thirty‐nine monolithic 3Y‐PSZ crowns were milled from pre‐sintered zirconia discs, using the InLab MC X5 equipment (Dentsplay Sirona, Bensheim, Germany) with three different milling protocols (*n* = 13): (S) slow, (N) normal, and (F) fast. The difference between the protocols is the speed and time required to produce the crowns, and the burs milling path (Table 1) [15]. The same diamond‐coated burs were used for all protocols: Bur 0.5 ZrO, Bur 1.0 ZrO, and Bur 2.5 ZrO (Dentsply Sirona, Bensheim, Germany). Two pre‐sintered zirconia discs were used to produce all crowns (total of 20 crowns per disc, with 6 to 7 crowns of each protocol per disc), and a new set of burs was used for each disc.

**TABLE 1:** Experimental groups and description of the CAD/CAM milling protocols.

Groups	Protocols	Mode	Time
S	Slow	Refined	25 min per crown
N	Normal	Conventional	18 min per crown
F	Fast	Fast	12 min per crown

After dry milling, crowns were carefully detached from the ceramic disc and sintered in the inLab Profire furnace (Dentsply Sirona, Bensheim, Germany). The sintering cycle was performed at 1450°C for 80 min, according to the YZ HT Speed protocol recommended by the manufacturer.

### 2.2 Adaptation Analysis

The adaptation was evaluated by measuring the gap thickness between crown and abutment, using the replica technique [11, 29]. Abutments and crowns were cleaned, and an extra light viscosity addition silicon impression material (Panasil Initial Contact X‐Light, Kettenbach, Huntington Beach, CA, USA) was inserted into the inner surface of the crown that was placed over the abutment. A static load of 750 g was applied to the occlusal surface of the crown for 5 min, to standardize the time and pressure during the polymerization of the impression material.

After removing the crown from the abutment, with the impression material simulating the cement layer, the excess of material was removed from the margins with a surgical blade. The space occupied by the abutment was filled with a light viscosity impression material of a different color (Panasil Initial Contact Light, Kettenbach, Huntington Beach, CA, USA), to obtain a consistent structure that could be removed from the interior of the crowns. An extra layer of impression material (Panasil Initial Contact Light, Kettenbach, Huntington Beach, CA, USA) was also added to the external surface of the replica to avoid damaging it during the cutting process.

The final replica constituted by an extra light silicone (representing the cement layer) and a light silicone (representing the abutment and crown) was cut with a surgical blade, in the mesio‐distal (M‐D) direction, into two equal parts (hemi‐sections), to analyze the thickness of the extra light silicone layer [11, 29]. Only the M‐D sections were analyzed as crowns were symmetrical.

Images were obtained from the hemi sections with a camera (EOS Rebel T5i, Canon, Tokyo, Japan) and were evaluated with image processing software (ImageJ Launcher, National Institutes for Health, Bethesda, USA). Standardized images were obtained by fixing the distance between the camera and the replica using a tripod, maintaining a constant magnification, and including a calibration scale [11].

The cement gap thickness (extra light silicone thickness) was measured in five predefined regions [11, 29, 30]: (M) marginal; (GA) gingival‐axial angle; (A) axial; (AO) axio‐occlusal angle; (O) occlusal. One trained and calibrated operator performed all the measurements. Measurements were performed on both sides (mesio and distal) of the hemi‐section and the average value of each region for each crown was used in the statistical analysis.

### 2.3 Marginal Quality Analysis

Marginal quality was evaluated using a qualitative score [20]. Each crown was examined in a light stereomicroscope (Zeiss Stemi 2000‐C, Carl Zeiss Microscopy GmbH, Göttingen, Germany) equipped with a camera (AxioCam ERc 5s, Carl Zeiss Microscopy GmbH, Göttingen, Germany). Crowns were placed in the horizontal position, and images of the margins (mesial, buccal, distal, and lingual regions) were obtained at 10× and 20× magnification. Images were analyzed by two previously trained blind examiners. The number and severity of the margin defects were graded according to a severity scale with scores from 1 to 5 [20]. The intra and inter‐examiner agreement was greater than 0.80 (Cohen's Kappa).

### 2.4 Occlusal Quality Analyses

For the qualitative analysis of the anatomical characteristics, images of the crowns occlusal surface were obtained using a light stereomicroscope (Zeiss Stemi 2000‐C, Carl Zeiss Microscopy GmbH, Göttingen, Germany) equipped with a camera (AxioCam ERc 5 s, Carl Zeiss Microscopy GmbH, Göttingen, Germany), at 8× magnification.

For the quantitative analysis, the occlusal dimensional discrepancy between the different protocols was assessed. First, crowns were placed in the respective abutments, scanned with an intraoral scanner (3Shape Trios 3; 3Shape, Copenhagen, Denmark) and the digital models were converted into STL files [13, 19]. Files were processed using Geomagic Wrap 2021 software (3D system, Rock Hill, SC, USA). A pair of crowns, reference and test, were aligned, and the occlusal area was set as the region of interest for the analysis. A crown from group N was used as a reference and compared with crowns from groups S and F (N vs. S, N vs. F). Subsequently, comparisons were made between groups S and F (S vs. F), using a crown from group S as reference.

The values (mm) of total mean distance, positive mean distance (distance above the reference occlusal surface), and negative mean distance (distance below the reference occlusal surface) were obtained.

### 2.5 Statistical Analyses

Gap thickness data passed the Shapiro–Wilk normality test (*p* > 0.05). For each region evaluated, one‐way ANOVA and Tukey's test (*α* = 0.05) were used to compare the gap thickness between the three experimental groups. Margin severity scores were analyzed, for each region separately, with Kruskal–Wallis and Student–Newman–Keuls tests (*α* = 0.05). The occlusal discrepancy data passed the Shapiro Wilk normality test (*p* > 0.05) and were analyzed with ANOVA and Tukey's test (*α* = 0.05).

## 3 Results

### 3.1 Adaptation Analysis

**FIGURE 1:**
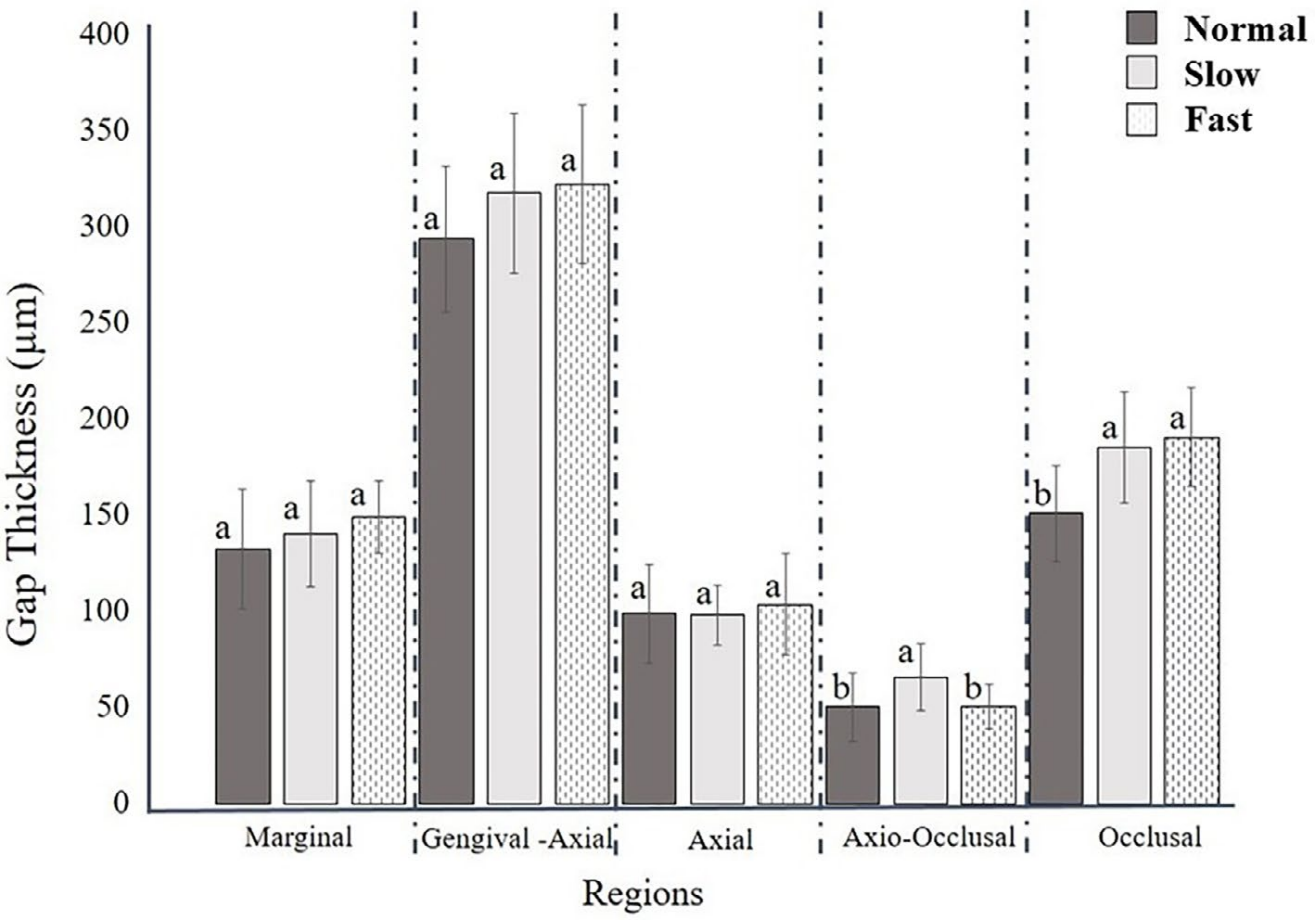
Mean and standard deviation (error bar) values of the gap thickness (in μm) in the different measurement regions for the three experimental groups. For the same measurement region, similar letters indicate statistically similar values (*p* > 0.05).

Figure 1 shows the mean values and standard deviation of the gap thickness (in μm) in the different measurement regions for the three experimental groups. There were no significant differences between groups for gap thickness in the marginal (*p* = 0.316), gingival‐axial angle (*p* = 0.189), and axial (*p* = 0.821) regions. In the axio‐occlusal angle (*p* = 0.033) and in the occlusal region (*p* = 0.002), there were statistical differences between the groups. The S protocol resulted in greater gap thickness in the axio‐occlusal angle and occlusal regions than the N protocol (*p* < 0.05). The protocol F showed similar gap thickness to N in the axio‐occlusal angle, but it was greater in the occlusal region.

### 3.2 Marginal Quality Analysis

**FIGURE 2:**
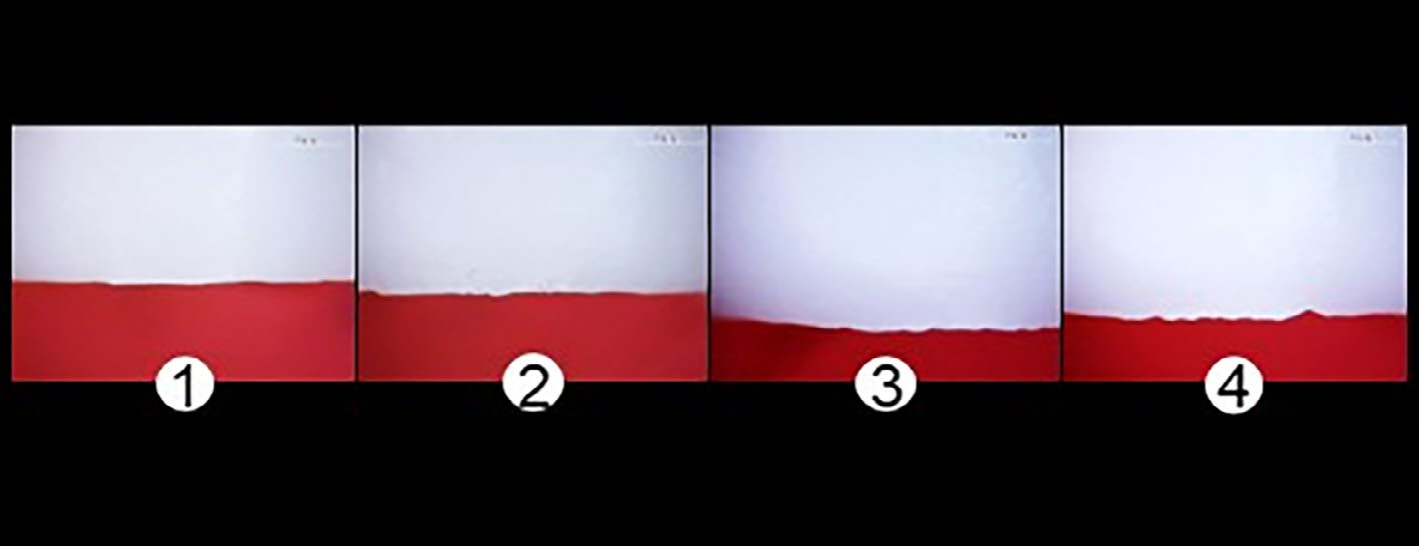
Representative images of the margin severity scores observed for the experimental groups: [13] score 1—smooth edge, no defects; score 2—smooth edge, few, small separate defects; score 3—several small defects; score 4—rough edge, few large defects.

Representative images of the margin severity scores identified for the experimental groups are presented in Figure 2. It was possible to identify scores ranging from 1 to 4. Score 5, which indicates a rough edge with several large defects, was not observed, and the frequency of score 4 was low. There was no difference between the groups for the total margin severity score (*p* > 0.05), which was calculated considering all marginal regions evaluated in each crown (Table 2). Yet, when the scores for each region were analyzed separately, group F resulted in higher severity scores than groups N and S in the mesial (*p* < 0.001) and buccal (*p* = 0.015) marginal regions.

**TABLE 2:** Median marginal severity scores and interquartile range (Q1; Q3) for the experimental groups.

Group	Distal	Lingual	Mesial	Buccal	Total margin
N	3 a (2; 3)	1 a (1; 1)	2 b (2; 2)	1 b (1; 1)	1 a (1; 2)
S	2 a (2; 2)	1 a (1; 1)	2 b (2; 2)	1 b (1; 1)	2 a (1; 2)
F	3 a (2; 3)	1 a (1; 1)	3 a (2; 3)	1 a (1; 2)	2 a (1; 3)
p	0.082	0.846	< 0.001	0.015	0.102

*Note:* Median values followed by the same letter in the same column are statistically similar (*p* > 0.05).

### 3.3 Occlusal Quality Analyses

**FIGURE 3:**
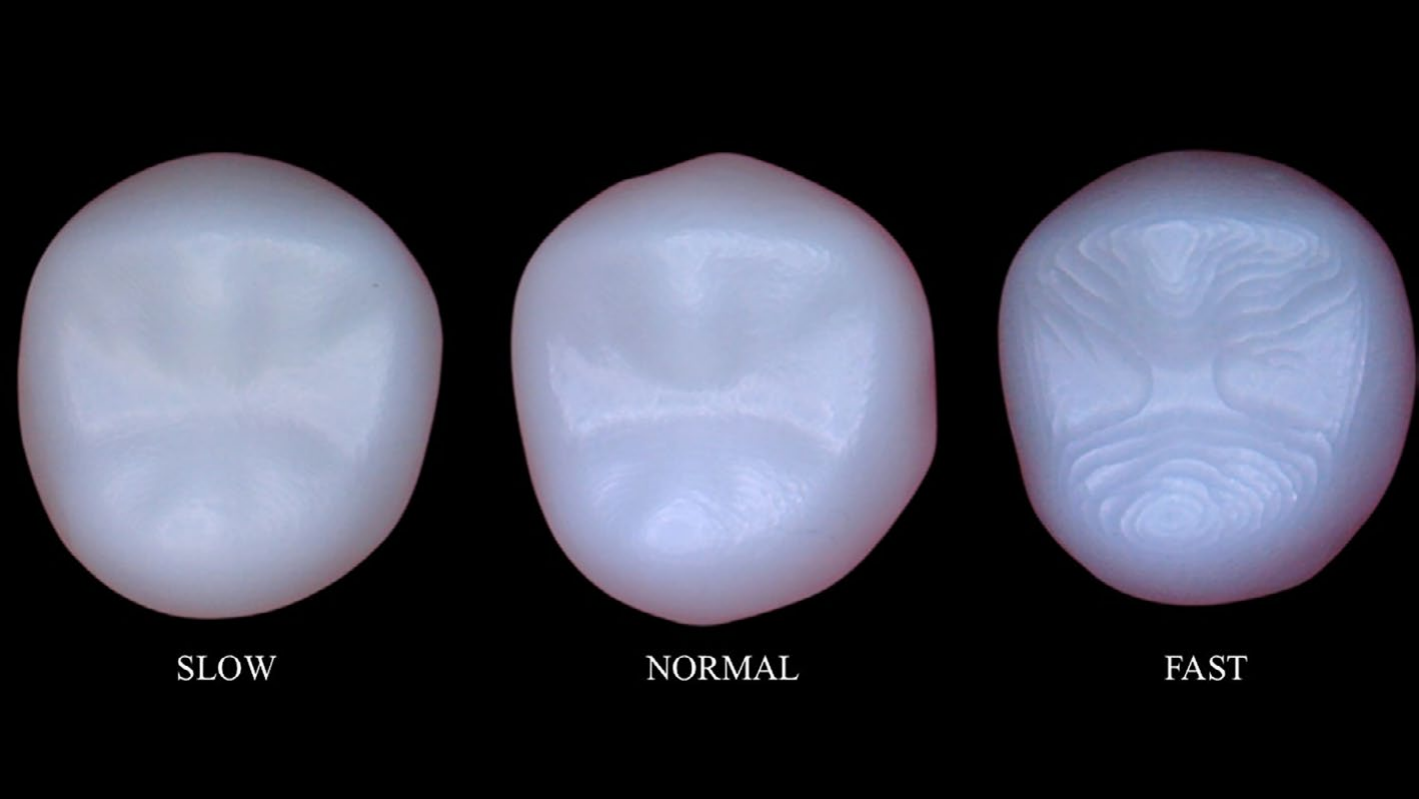
Representative images of the occlusal surfaces of monolithic zirconia crowns milled with the slow (S), normal (N), and fast (F) protocols.

Qualitative analysis indicated that the type of milling protocol resulted in distinct occlusal characteristics of the monolithic crowns (Figure 3). Crowns milled with the normal (N) and slow (S) protocols showed similar and high‐quality occlusal features. Group S had a more uniform surface and refined anatomical details, especially when compared to group F. The occlusal anatomy of crowns milled with the F protocol was heterogeneous, showing visible marks produced by the milling path of the CAD/CAM burs and a lack of anatomical details.

**FIGURE 4:**
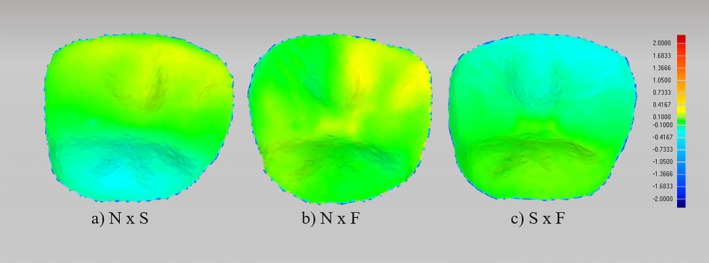
Color maps of the dimensional discrepancy of the occlusal surface according to each comparison: (a) N vs. S; (b) N vs. F; (c) S vs. F. Red indicates a positive error, blue indicates a negative error and green indicates good match.

In the quantitative analysis, there were no differences for the negative and positive occlusal mean distance between groups (*p* > 0.05) (Table 3, Figure 4). For the total mean discrepancy, the distance between the occlusal surface of crowns from groups F and N (reference) was similar to the distance observed between groups S and N. Crowns produced with the F protocol had a greater total mean distance in comparison to group S than group N (*p* < 0.05).

**TABLE 3:** Mean values (standard deviation) of the total, positive, and negative distance between the occlusal surface of the crowns milled with different protocols.

	Occlusal discrepancy (mm)		
	Total	Positive	Negative
N vs. S	−0.56 (0.01) ab	0.067 (0.025) a	−0.815 (0.059) a
N vs. F	−0.54 (0.03) a	0.074 (0.042) a	−0.879 (0.168) a
S vs. F	−0.57 (0.02) b	0.072 (0.028) a	−0.762 (0.071) a

*Note:* Mean values followed by the same letter in the same column are statistically similar (*p* > 0.05).

## 4 Discussion

The CAD/CAM technology is in constant evolution, providing more efficient processes for the design and production of dental prosthesis, tailoring the treatment to the patients' clinical needs [8, 9]. The development of open systems that allow the dentist and the laboratory technician to choose among different milling protocols to produce the restorations is an important improvement in the technology [9, 14, 15]. Therefore, this study focused on characterizing the adaptation and anatomical quality of monolithic zirconia crowns produced with these novel milling protocols.

The gap thickness of the monolithic crowns was affected by the milling protocol, accepting the first study hypothesis. Crowns milled with the normal protocol (group N) had smaller gaps in the axio‐occlusal angle and occlusal regions. The slow protocol was longer, meaning that the burs spent more time milling and refining the restoration surfaces, which led to greater gap thickness in the inner occlusal regions of the crowns, as more material was removed. In addition, milling the internal occlusal area of dental crowns can be very challenging due to limitations of the shape and diameter of the burs, which could also explain a greater gap at the occlusal surface for the fast protocol in comparison to the normal one.

Yet, the gap thickness in the marginal, gingival‐axial angle, and axial regions of the zirconia crowns was similar among the milling protocols, but greater than the ones reported in clinical investigations [24, 25]. Direct comparison between gap values obtained in different in vivo and in vitro studies is limited by several factors, including the type of prosthesis and abutment design, cement space thickness, scanning process, CAM milling unit, restorative material, and methodology used to evaluate the prosthesis adaptation [10, 11, 13, 17, 20, 24, 25, 29, 30]. In the present study, a cement space thickness of 80 μm resulted in gap values above 300 μm in some regions, despite carefully following all clinical protocols and manufacturer recommendations, suggesting that a smaller cement space could be desired for clinical application. Moreover, previous studies that investigated the adaptation of ceramic restorations reported larger gaps for CAD/CAM technology in comparison to other manufacturing methods [10, 11, 24, 29], mainly due to limitations of the milling tools in reproducing the internal area of small restorations, such as the maxillary premolar crown used in the present study.

The total margin severity score was similar among groups, and a low frequency of severe defects was identified. These findings indicate that the milling protocols were carefully designed to guarantee a good marginal quality, regardless of the time required to produce the restorations. Nevertheless, the manufacturer does not provide detailed information on the protocols' milling path, which limits the study discussion. Yet, it is important to highlight that zirconia CAD/CAM discs were machined in a partially sintered condition. Defects and micro‐cracks introduced during the milling process could be minimized by the final sintering cycle due to the increase in the material's density and sintering shrinkage [16]. A previous study reported similar flexural strength between CAD/CAM milled and polished 3Y‐PSZ specimens, and no effect of the milling protocol on their mechanical behavior and crystal phase distribution as all treatments were performed in a pre‐sintered stage [14]. In addition, the high‐toughness and high‐strength second‐generation 3Y‐PSZ investigated has high edge chipping resistance [1, 2, 4, 6, 7]. Another investigation also reported that the chamfered finish line preparation, as the one used in the present study, could result in crowns with smoother margins and fewer defects than other preparations [19].

However, when the different regions of the margin were analyzed separately, the mesial and buccal regions of the crowns produced by the fast protocol presented greater severity scores than the other protocols, meaning that more severe defects were identified. Therefore, the second study hypothesis was accepted. Fast milling has limitations when reproducing more refined angles and shapes, as also observed for the occlusal surface of the crowns. In the present study, crowns were not subjected to any type of adjustment or surface treatment before the analyses. Clinically, pre‐ and post‐sintering finishing and polishing protocols could be performed by the laboratory technician and/or dentist in order to improve the dental prosthesis surface quality [8, 34].

Qualitatively, it was possible to identify distinct occlusal surface characteristics for crowns produced with the different milling protocols, accepting the third study hypothesis. Slow and normal protocols produced crowns with good anatomical details and only small differences were perceived, mostly related to a greater surface finish for the slow protocol. On the contrary, anatomical and surface characteristics produced by the fast protocol were less refined. In this case, major adjustments and finishing protocols would be required to produce a clinically acceptable monolithic prosthesis [35]. These findings corroborate with previous investigations that characterized the topography of 3Y‐PSZ discs and monolithic crowns produced with the slow, normal, and fast milling protocols [14, 15].

When the dimensional occlusal discrepancy was quantitatively analyzed, it was not possible to identify significant differences between the occlusal surface of crowns produced by the slow and fast protocols in comparison to the normal one, which is a positive finding. Overall, a good match was found between the distinct occlusal surfaces. Yet, it should be acknowledged that the scanning and image processing steps may have reduced the accuracy of the measurements, leading to high discrepancy values, and small differences may not be detected [13, 19]. Extrapolation of the study findings should also consider that only one type of CAD/CAM equipment and ceramic material were investigated, and that all gap thickness measurements were performed in the same master abutment.

The slow and normal protocols produced monolithic zirconia crowns with similar adaptation, marginal, and occlusal quality, despite the minor differences in gap thickness and anatomical characteristics. The literature also reported similar fracture load values after mechanical aging for 3Y‐PSZ crowns produced with the slow and normal protocol [15], supporting their clinical application. The fast‐milling protocol resulted in inferior anatomical occlusal and marginal characteristics, meaning that extra polishing or veneering steps would be necessary to obtain clinically acceptable restorations.

## 5 Conclusion

Within the limitations of this current study, it was concluded that the type of CAD/CAM milling protocol affected the adaptation, marginal and occlusal quality of monolithic zirconia crowns. Crowns produced using the normal milling protocol had smaller gaps in the occlusal region, and similar marginal and occlusal quality than crowns produced with the slow protocol. The fast‐milling protocol resulted in crowns with reduced marginal integrity in the mesial and buccal regions and less refined occlusal anatomy in comparison to the other protocols.

## Funding

This work was supported by Fundação de Amparo à Pesquisa do Estado do Rio Grande do Sul (19/2551‐0000677‐2) and the Conselho Nacional de Desenvolvimento Científico e Tecnológico (302587/2017‐9).

## Disclosure

The authors have nothing to report.

## Conflicts of Interest

The authors declare no conflicts of interest.

## Data Availability

The data that support the findings of this study are available from the corresponding author upon reasonable request.
